# Asymptomatic myocardial metastasis from cancers of upper aero-digestive tract detected on FDG PET/CT: a series of 4 cases

**DOI:** 10.1186/1470-7330-14-16

**Published:** 2014-04-28

**Authors:** Ameya D Puranik, Nilendu C Purandare, Sheela Sawant, Archi Agrawal, Sneha Shah, Prafful Jatale, Venkatesh Rangarajan

**Affiliations:** 1 Department of Nuclear Medicine and Molecular Imaging, Tata Memorial Hospital, Dr E Borges Marg, Mumbai 400012, India; 2 Department of General Medicine, Tata Memorial Hospital, Dr E Borges Marg, Mumbai 400012, India

**Keywords:** Head neck, Esophagus, Asymptomatic, Myocardial metastasis, FDG PET, CT

## Abstract

Metastatic involvement of the heart is a rare occurrence and remains undiagnosed until autopsy. In some instances, patients may have cardiac symptoms, leading to ante-mortem diagnosis. Although most primary cancers have been documented to metastasize to heart, the existing literature on cancers of upper aero-digestive tract is an exception, with only a few reports. We report four cases of upper aero-digestive tract cancers, three of which arise from oral cavity, one from lower esophagus, metastasising to the myocardium, detected on ^18^ F – Fluoro-deoxy-glucose Positron Emission Tomography/Computed Tomography (FDG PET/CT) study , in the absence of related symptoms.

## Background

Myocardial metastases have been reported from almost all cancers, commonest of these being lung, breast and melanoma primaries. Upper aero-digestive tract cancers have shown extremely low incidence of metastases to heart; the reasons being a lack of prospective imaging work-up and absence of whole body imaging. Also, imaging work-up so far was prompted only after the occurrence of symptoms; thus the larger subset of asymptomatic patients was left uninvestigated. We therefore report a series of four cases, one being a primary from GE junction and three from head neck region, wherein myocardial metastases were detected on whole body FDG PET/CT imaging. These patients were referred for PET/CT imaging for established indications; staging of esophageal cancer and restaging of head neck cancers following a biopsy proven local site recurrence. Thus, FDG PET/CT was obviously being done, with the future plan of treatment in view, and hence detection of myocardial metastases in all these four patients had a significant impact on management, especially since all were clinically asymptomatic. This work not only highlights the role of fusion imaging with PET/CT in picking up rare distant metastatic sites and changing management, but also makes the clinicians aware of the possibility of myocardial metastases from cancers of upper aero-digestive tract.

## Case presentation

### Case 1

**Figure 1:**
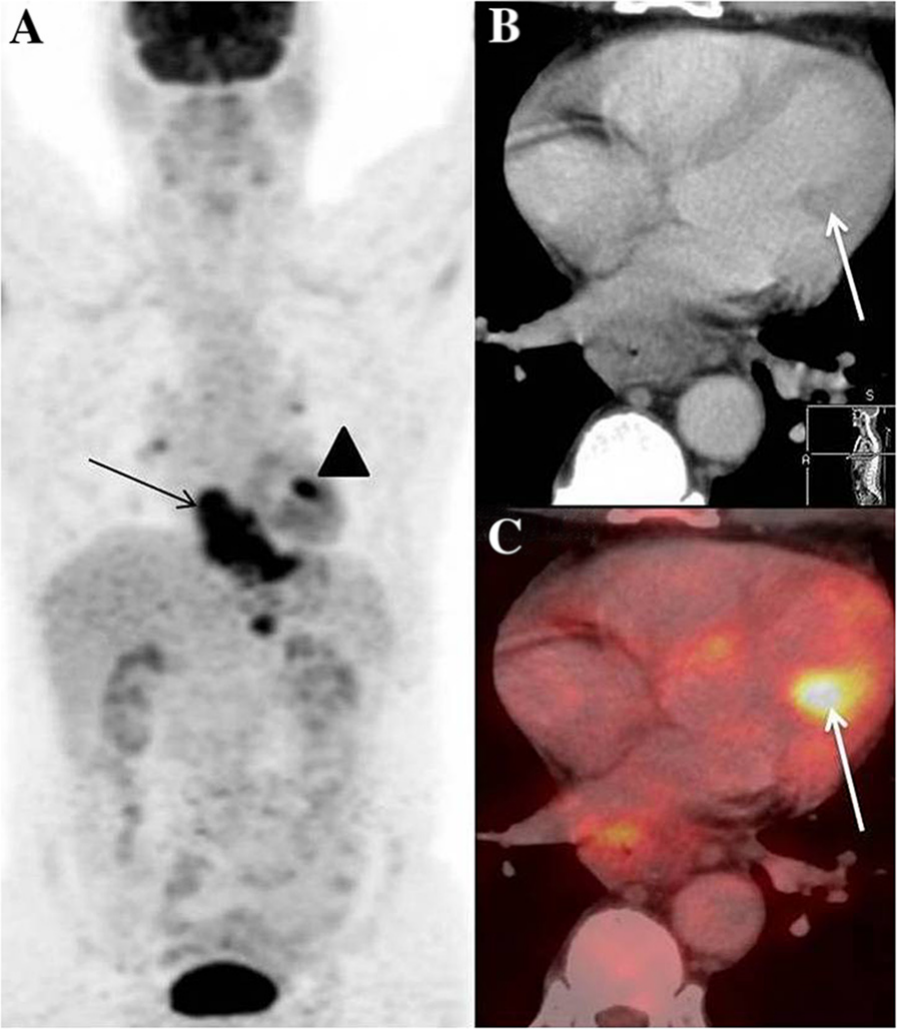
**Myocardial metastases from esophageal cancer. A**- MIP image showing primary esophageal mass (arrow) with focal uptake in region of heart (arrowhead), **B**- Hypodense lesion in left ventricular myocardium (arrow) on axial CT images showing, **C**- FDG uptake on axial fused PET/CT images (arrow).

**Figure 2:**
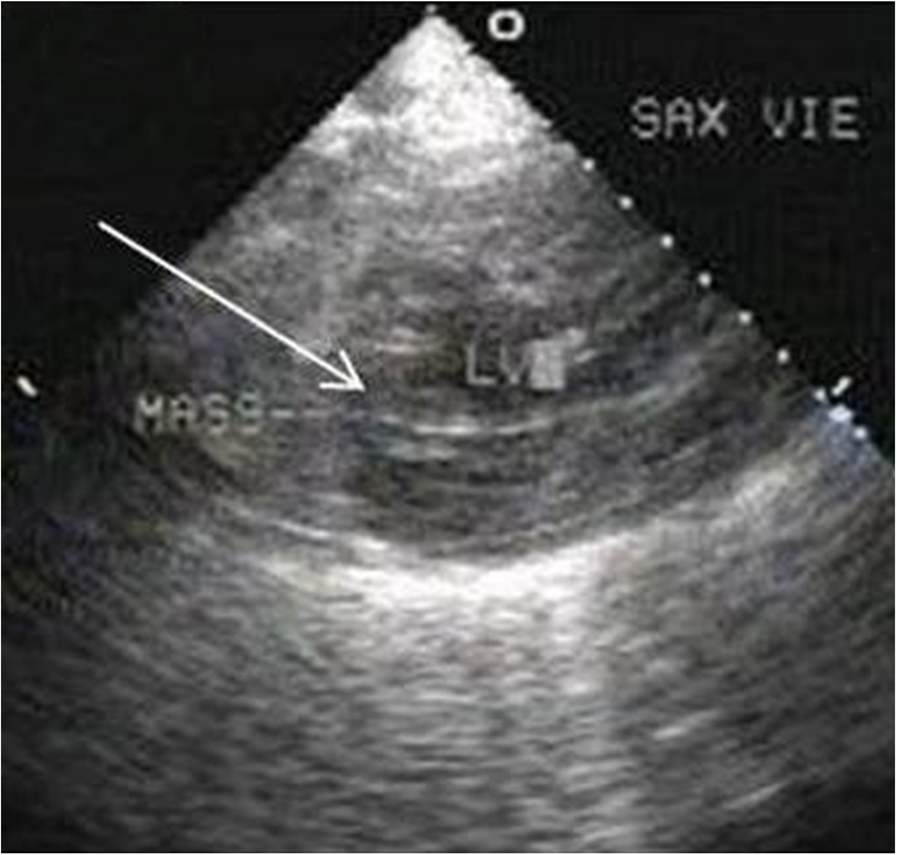
2-D Echo image showing mass in left ventricular myocardium (arrow).

A 53 year old male presented to our institution with progressive dysphagia since 2 months. Upper gastro-intestinal endoscopy showed an ulcero-proliferative mass involving the lower third of esophagus and gastro-esophageal junction (GEJ). Biopsy showed adenocarcinoma cells. Subsequently he underwent a whole body ^18^ F –FDG PET/CT study for pre-operative staging. MIP image showed tracer concentration at the primary site in lower esophagus and GEJ (Figure 1A - arrow), and in upper abdominal nodes. Focal tracer uptake was noted in cardiac region (1A - arrowhead), which on axial CT (1B – arrow) and fused PET/CT (1C – arrow) images was localized to the left ventricular myocardium. This was initially thought to be physiological FDG uptake in papillary muscle, hence 2-D Echo was done for confirmation. 2-D Echo showed a mass in the left ventricular myocardium (Figure 2 - arrow) confirming the presence of myocardial metastases. The management intent changed to palliative, hence no further PET/CT imaging was done for follow up.

### Case 2

**Figure 3:**
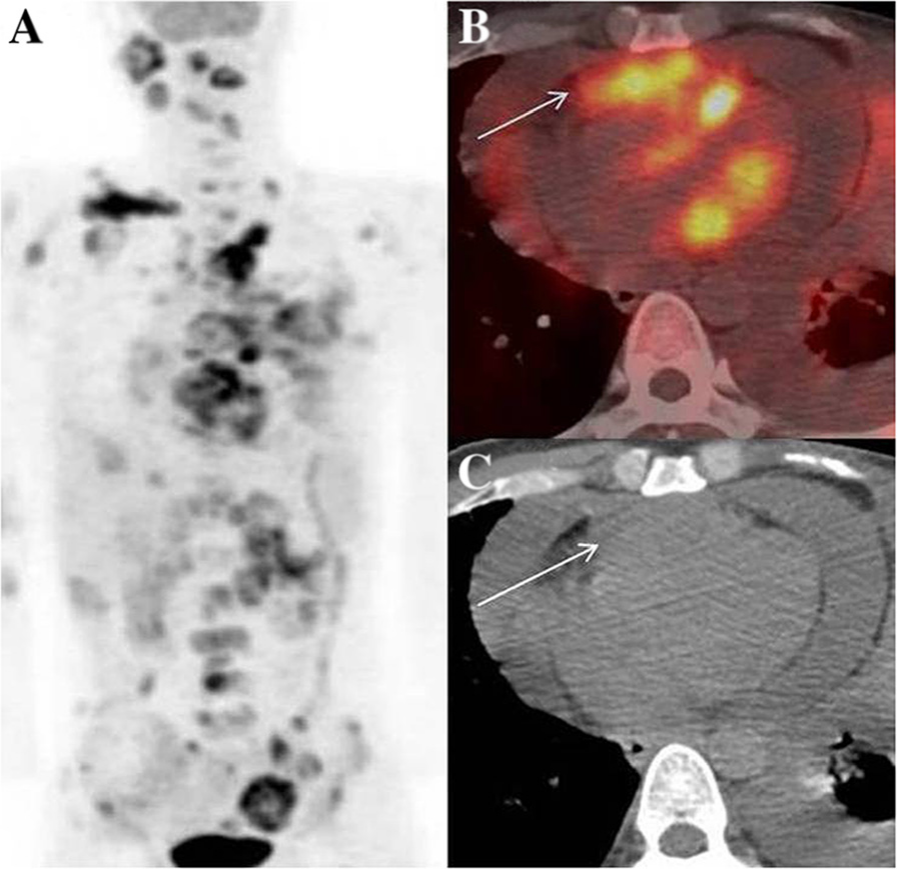
**Myocardial metastases from buccal mucosal cancer. A** - MIP image showing multiple metastatic foci, **B**- Axial fused PET/CT image showing tracer uptake in the cardiac region (arrow), which corresponds to **C** -hypodense lesion on axial CT image (arrow).

**Figure 4:**
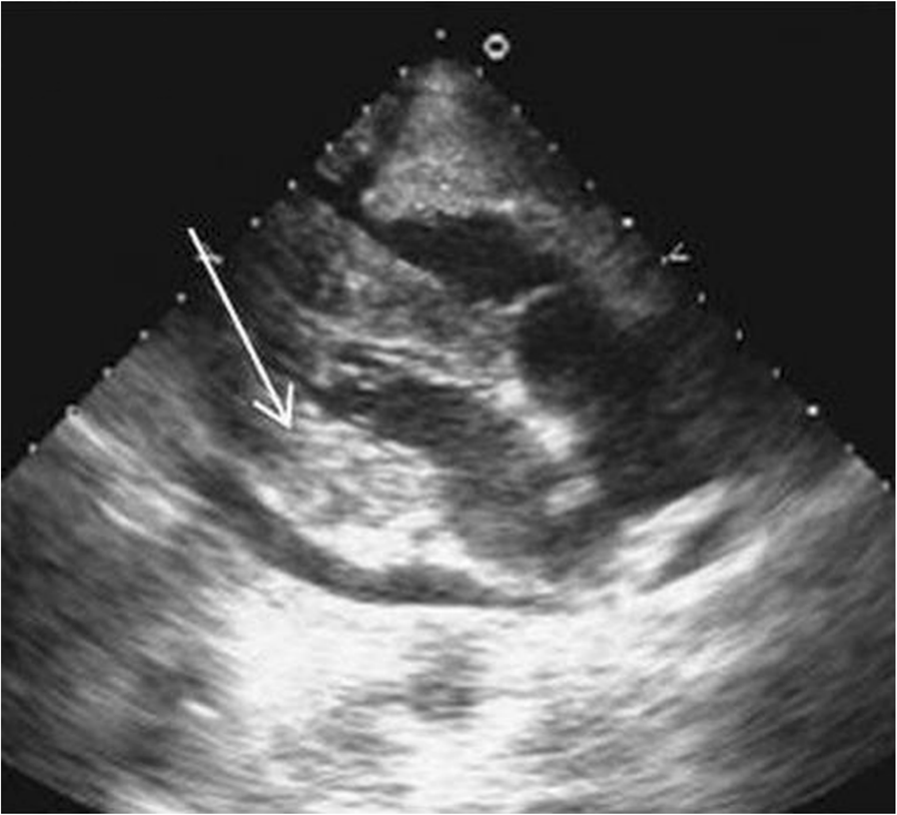
2-D Echo image showing left ventricular myocardial mass (arrow).

A 29 year old male, a tobacco chewer since 8 years, was diagnosed with cancer of left buccal mucosa. He was treated with chemo-radiation. 2 months after treatment completion, follow up PET/CT was unremarkable. He later presented with significant weight loss and clinically palpable cervical nodes, after a disease free interval of 18 months. Biopsy revealed recurrent squamous cell cancer. Whole body FDG PET/CT study was done for restaging. MIP images showed multiple foci of uptake in neck region, suggestive of local site recurrence and metastatic nodes (Figure 3A). Also seen were metastatic foci in bones, liver and other nodal regions. A discrete focal uptake in cardiac region was seen as an FDG avid hypodense area on axial fused PET/CT (3B –arrow) and CT (3C – arrow) images, which was highly suspicious for metastases. 2-D Echo was performed for cardiac status evaluation, before starting palliative chemotherapy. It confirmed the presence of mass in the left ventricular myocardium (Figure 4 - arrow). Patient was not imaged further and eventually lost to follow-up.

### Case 3

**Figure 5:**
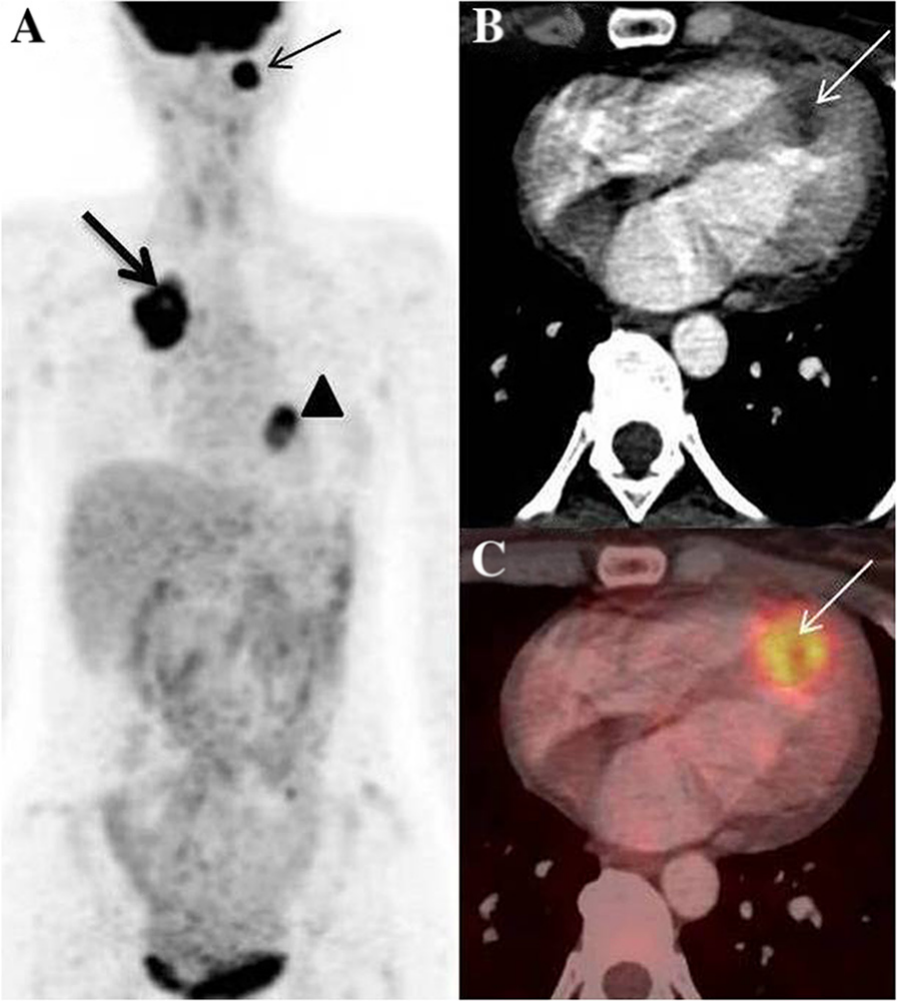
**Myocardial metastases from tongue cancer. A** - MIP image showing tracer uptake in left neck region (thin arrow), with another large area in thorax (thick arrow). Focal uptake is noted in cardiac region (arrow-head). **B** - large hypodense lesion in left ventricular myocardium, **C** - FDG uptake in the myocardial mass.

**Figure 6:**
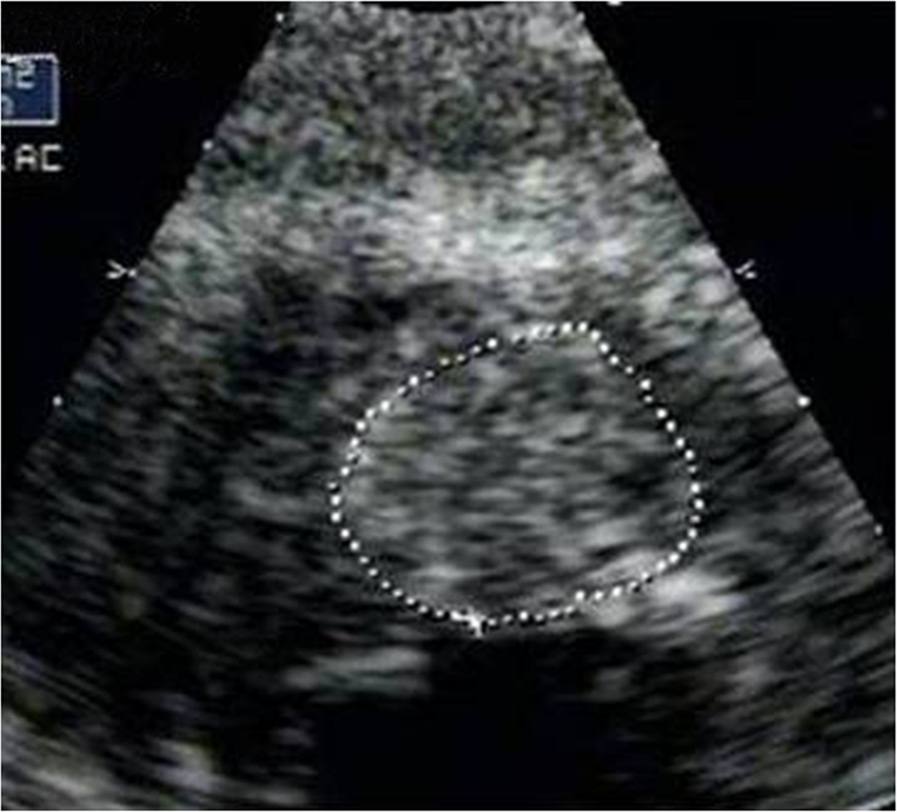
2-D Echo image showing large mass in left ventricular myocardium (arrow).

A 32 year old female patient was a treated case of squamous cancer of right lateral border of tongue. She underwent wide excision and right lateral neck dissection, followed by a disease free interval of 2 years. She then presented with a swelling over ala of the nose, biopsy of which revealed metastatic squamous carcinoma from the known primary. Whole body PET/CT done for restaging showed focal uptake at the site of recurrence in neck region (Figure 5A - arrow), with a metastatic lung lesion (5A – bold arrow) and discrete intense tracer concentration in the region of heart (5A – arrowhead). Axial CT (5B – arrow) and fused PET/CT (5C – arrow) images showed a large hypodense FDG avid mass in the left ventricular myocardium. 2 D Echo performed for pre-chemotherapy work-up showed a 17 mm sized mass in the left ventricular myocardium adjoining the inter-ventricular septum (Figure 6 - arrow), confirming the presence of cardiac metastases. Patient was on palliative chemotherapy, with follow up CT scan at 3 months showing stable disease.

### Case 4

**Figure 7:**
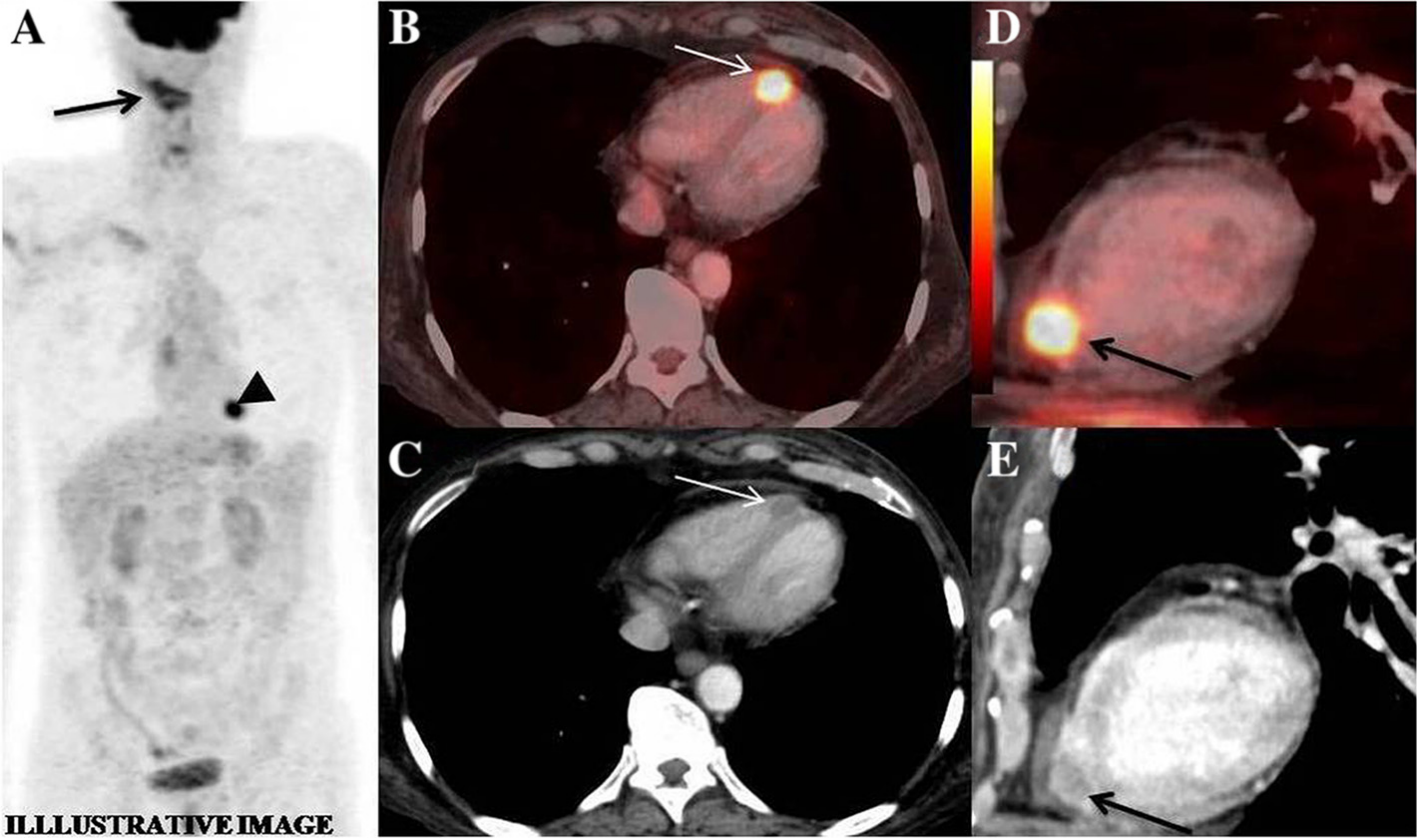
**(Image for article illustration). A** - MIP image showing tracer uptake in neck region (arrow) and focal uptake in the midline in chest (arrow-head), **B** &**C** - hypodense lesion in right ventricular myocardium along the septum on axial CT (arrow) and fused PET/CT (arrow) image, **D** &**E** - same lesion seen on sagittal CT (arrow) and fused PET/CT (arrow) image.

A 46 year old gentleman, case of squamous cancer of right vallecula, treated with chemo-radiation came for follow-up at one year. Pan-endoscopy revealed suspicious thickening in right vallecula and adjacent base of tongue. Biopsy revealed squamous cells. Patient underwent a whole body FDG PET/CT study for restaging. MIP images showed tracer uptake in the right neck region (Figure 7A - arrow). Discrete intense focus of FDG uptake was seen in the region of heart (Figure 7A - arrowhead). Axial fused PET/CT images (Figure 7B - arrow) showed that the focus corresponded to myocardium overlying the right ventricle, adjacent to the inter-ventricular septum, appreciated better on sagittal images (Figure 7D - arrow). Contrast enhanced axial (Figure 7C - arrow) and sagittal CT (Figure 7E - arrow) images showed a hypodense area, corresponding to the site of FDG uptake in the myocardium overlying the right ventricle, thus confirming it to be metastasis. 2-D Echo was confirmatory for a mass in right ventricular myocardium. Since patient had distant metastases, surgery was ruled out and he was started on palliative chemotherapy. Patient was referred to a peripheral cancer centre for logistic reasons and hence lost to follow up.

## Discussion

Metastatic cancer involving the heart was first described by Boneti in 1700; the diagnosis being made post-mortem [1]. The first report of ante-mortem diagnosis was published in 1913 [2]. Cardiac involvement from a metastatic cancer can be by direct extension, by hematogenous or lymphatic route or by intra-cavitary diffusion either by inferior vena cava or by the pulmonary veins [3]. Lymphatic spread tends to give rise to pericardial metastases, as seen in lung and breast cancer, owing to their topography; whereas hematogenous spread leads to myocardial metastases [4]. Due to their propensity for generalized hematogenous spread, malignant melanomas frequently metastasise to the heart [5]. Esophageal cancers metastasise to the heart predominantly by lymphatics. Tumor cells infiltrate the mediastinal lymph nodes, through which they reach the epicardial and then the myocardial lymphatic system [6]. However, all reports of esophageal cancers with this spread pattern are of squamous cell origin [7-11], unlike Case 1 where adenocarcinoma cells were seen on histology. Rare occurrence of myocardial metastases has been reported from primary head and neck cancers. DeLoach and Haynes in 1953 discovered on autopsy that one of the nine patients of primary malignancy of tongue had myocardial or pericardial involvement [12]. Gassman et al. reviewed 4124 autopsies of cancer patients, of which 126 had primary tongue tumors. 2 (1.5%) of these patients with tongue primary cancer had myocardial involvement [13]. To the best of our knowledge, there are six reports in 7 patients, with cardiac metastases from head and neck primary cancers; 6 of which were detected ante-mortem, one on autopsy; [14-19]. The above report shows that primary carcinoma of tongue metastasizes to heart with some degree of frequency, with no explanation for this, being offered so far. Primary origin in vallecula, in Case 4, though a new site of primary, its anatomical proximity to base of tongue, should also be taken note of. In case 2, the primary cancer is in the buccal mucosa, which is an altogether unique location for head and neck primary with myocardial metastases.

Detection of cardiac metastases in most of the reports is by Echocardiography or Cardiac Magnetic Resonance Imaging (MRI). Echocardiography, particularly 2D imaging, is sensitive for detection of cardiac metastases [20]. 3 out of the 4 cases in our series have an echocardiographic confirmation. FDG PET/CT with its ability for whole body fusion imaging is used for detection of distant metastases in most of the cancers, including esophageal cancers. Oncological FDG PET/CT is performed with a fasting period of 6 hours, ensuring minimum myocardial FDG uptake [21]. Hence, in case of cardiac metastases, the mechanism of increased FDG uptake at that site is GLUT receptor over-expression, as in the primary site. Reports of cardiac metastases on PET/CT are seen in melanoma [22], Ewings tumor [23], renal cell carcinoma [24] and breast cancer [25], to name a few, with no reports from cancers of upper aero-digestive tract. FDG PET/CT is routinely used for restaging of head and neck cancers [26]. Also, since dual modality imaging is possible with PET/CT, CT component of PET/CT has been used for localization and confirmation of tracer uptake in myocardium [27]. All existing reports of myocardial metastases in head neck cancers are in patients who showed local disease control and on follow up presented with symptoms of chest pain, angina or syncopal episodes, for which further evaluation was done, which led to detection of cardiac metastases. Contrary to this, all patients in our series were asymptomatic. PET/CT studies were done in these patients for restaging, due to clinical suspicion of local recurrence; with the intent to rule out distant metastases. In addition, 2-D Echocardiography was done for confirmation of metastatic involvement (Case 1 and 4). In patients with extensive metastases (Case 2 and 3), cardiac status evaluation was done as a part of routine pre-chemotherapy work-up, which in retrospect, helped us confirm PET/CT imaging findings.

Cardiac involvement by metastatic malignancy is seen exclusively in the setting of widespread metastatic disease; the recent autopsy series showing 5% of 2833 cadavers having metastatic disease from malignant neoplasms with myocardium being one of the sites [28]. Case 4 in our series, showed local recurrence in vallecula, with an isolated site of distant metastases in myocardium; in the absence of nodal disease. This is an highly unusual phenomenon which changed the intent of management from potentially curative radical salvage to palliative care.

Cardiac metastases from upper aero-digestive tract cancers is thus an uncommon occurrence with majority of cases detected on autopsy and otherwise, only a handful of symptomatic patients were diagnosed on imaging by echocardiography and most recently by cardiac MRI.

## Conclusion

In conclusion, whole body dual modality imaging with PET/CT in cancers of upper aero-digestive tract done for restaging can uncover such rare metastatic sites especially in asymptomatic patients, which otherwise would have been undiagnosed. It is a valuable addition to the existing literature on myocardial metastases, and also highlights the role of FDG PET/CT in detection of such rare sites which can very often have a significant impact on patient management.

## Methods

Formal approval was granted by the Hospital Ethics Committee (HEC) and Institutional Review Board (IRB) of Tata Memorial Hospital.

## Consent

Written informed consent was obtained from the patient for the publication of this report and any accompanying images.

## Abbreviations

18−F: ^18^Fluorine; FDG: Fluoro-deoxy-glucose; PET: Positron emission tomography; CT: Computed tomography; MIP: Maximum intensity projection; 2-D ECHO: Two dimensional echocardiogram.

## Competing interests

The authors declare that they have no competing interests.

## Authors’ contributions

ADP – First Author who conceptualized the study after adequate review of literature and collected the cases and images. NCP – provide the radiological assessment as far as the CT component of PET/CT was concerned. SSawant – conducted the 2-D Echocardiography procedures. AA – seconded the findings on PET images seen by first author and also assisted in data collection. SS – seconded the findings made by first author and Radiologist for both PET and CT images. PJ – clinical follow up of all 4 cases was undertaken and documented. VR – Reviewed the literature along with the first author and helped in collating the data and designing the manuscript. All authors read and approved the final manuscript.
